# Prognostic Trends and Current Challenges in Candidemia: A Comparative Analysis of Two Multicenter Cohorts within the Past Decade

**DOI:** 10.3390/jof9040468

**Published:** 2023-04-13

**Authors:** Caroline Agnelli, Thaís Guimarães, Teresa Sukiennik, Paulo Roberto Passos Lima, Mauro José Salles, Giovanni Luís Breda, Flavio Queiroz-Telles, Marcello Mihailenko Chaves Magri, Ana Verena Mendes, Luís Fernando Aranha Camargo, Hugo Morales, Viviane Maria de Carvalho Hessel Dias, Flávia Rossi, Arnaldo Lopes Colombo

**Affiliations:** 1 Division of Infectious Diseases, Department of Medicine, Escola Paulista de Medicina, Universidade Federal de São Paulo, São Paulo 04024-002, Brazil; 2 Hospital do Servidor Público Estadual de São Paulo, São Paulo 04039-000, Brazil; 3 Santa Casa de Misericórdia de Porto Alegre, Rio Grande do Sul 90050-170, Brazil; 4 Santa Casa de Misericórdia de São Paulo, São Paulo 01221-010, Brazil; 5 Universidade Federal do Paraná, Curitiba 81531-990, Brazil; 6 Hospital das Clínicas da Faculdade de Medicina USP (FMUSP), São Paulo 05403-010, Brazil; 7 Hospital São Rafael, Salvador 41253-190, Brazil; 8 Hospital Israelita Albert Einstein, São Paulo 05652-900, Brazil; 9 Hospital Erasto Gaertner, São Paulo 81520-060, Brazil; 10 Hospital Nossa Senhora das Graças, Curitiba 80810-040, Brazil; 11 Pathology Department, Laboratório de Microbiologia da Divisão de Laboratório Central, Hospital das Clínicas da Faculdade de Medicina USP (FMUSP), São Paulo 05403-010, Brazil

**Keywords:** candidemia, invasive candidiasis, mortality, prognosis, antifungal therapy

## Abstract

Candidemia remains a major public health challenge due to its high mortality rates, especially in developing countries. Monitoring epidemiological trends may provide insights for better clinical outcomes. This study aimed to describe trends in the epidemiology, therapeutic practices, and mortality in candidemia through a retrospective comparative analysis between two surveillance cohorts of all candidemic adults at eleven tertiary hospitals in Brazil, from 2010–2011 (Period I) versus 2017–2018 (Period II). A total of 616 cases were diagnosed, with 247 being from Period II. These patients were more likely to have three or more coexisting comorbidities [72 (29.1%) vs. 60 (16.3%), *p* < 0.001], had a prior history of in-hospital admissions more often [102 (40.3%) vs. 79 (21.4%), *p* = 0.001], and presented with candidemia earlier after admission, within 15 days (0–328) vs. 19 (0–188), *p* = 0.01. Echinocandins were more frequently prescribed [102 (41.3%) vs. 50 (13.6%), *p* = 0.001], but time to antifungal initiation [2 days (0–14) vs. 2 (0–13), *p* = 0.369] and CVC removal within 48 h [90/185 (48.6%) vs. 148/319 (46.4%), *p* = 0.644] remained unchanged. Additionally, many patients went untreated in both periods I and II [87 (23.6%) vs. 43 (17.4%), *p* = 0.07], respectively. Unfortunately, no improvements in mortality rates at 14 days [123 (33.6%) vs. 93 (37.7%), *p* = 0.343] or at 30 days [188 (51.4%) vs. 120 (48.6%), *p* = 0.511] were observed. In conclusion, mortality rates remain exceedingly high despite therapeutic advances, probably associated with an increase in patients’ complexity and suboptimal therapeutic interventions. Management strategies should be tailored to suit epidemiological changes, expedite diagnosis to reduce the number of untreated eligible patients and guarantee early antifungal initiation and source control.

## 1. Introduction

Candidemia remains the most prevalent invasive nosocomial fungal infection worldwide [1]. Its incidence varies globally from 0.33 to 6.51 episodes per 1000 admissions [1,2,3], representing a major public health burden due to its increasing frequency and high mortality rates [1,4,5,6]. An even worse scenario is expected among developing middle-income countries, not only due to a higher number of patients at risk, but also due to a scarcity of diagnostic and therapeutic resources, combined with difficulties to implement and comply with guidelines, undertrained personnel, and often overloaded centers, with limited access to proper critical care [7]. In Brazil, contrasting with most northern hemisphere countries, epidemiological studies have persistently shown exceeding crude 30-day mortality rates, reaching over 70% in patients admitted at intensive care units, despite improvements in the overall quality of patient care over time [6,8,9,10,11,12].

Nevertheless, 30-day mortality analyses alone may be an overly late and eventually inaccurate endpoint to represent the true contribution of candidemia for the final outcome of patients who usually already present risk factors for other events potentially leading to death [1,8]. Moreover, although a favorable prognosis cannot be determined by antifungal choice alone, many of such previous epidemiological studies were still performed during periods when echinocandins, the current initial treatment of choice [5,13,14], were neither as widely used, nor as readily available.

Aside from well-known prognostic factors related to the host and intrinsic fungal virulence mechanisms [15,16,17], modifiable therapeutic aspects such as prompt antifungal initiation combined with timely and effective infection source control, including central venous catheter removal, also play an essential role for better clinical outcomes [18,19,20,21,22], but strategies to ensure earlier interventions are still needed [22].

We sought to describe the latest trends in the epidemiology, therapeutic practices, and mortality among adults with candidemia across several tertiary hospitals in Brazil in order to identify new or persisting challenges, and shape practical measures that could further improve patient prognosis in similar developing settings.

## 2. Methods

### 2.1. Study Design, Patient Selection, and Data Collection

We performed a retrospective analysis comparing two multicenter laboratory-based surveillance cohorts including all consecutive adult patients ≥18 years of age with at least one peripheral blood culture yielding a *Candida* species diagnosed at eleven public and private tertiary hospitals from different regions of Brazil, during Period I: 2010–2011 versus Period II: 2017–2018. Primary outcome was to determine trends in all-cause mortality rates within 14 and 30 days from index candidemia. Secondary outcomes were changes over time in epidemiological characteristics and therapeutic practices.

A trained medical investigator from each center was responsible for data collection through a pre-established clinical form, using a dictionary of terms to assure consistency. The medical history and laboratory information of every patient were gathered up to 30 days from index candidemia, or death. The data used in this work were collected prospectively through a routine laboratory-based surveillance protocol, including: demographics, underlying medical conditions requiring active treatment or follow-up, department of admission at the time of the diagnosis of candidemia, risk factors and conditions associated with candidemia within the last 30 days, such as the use of broad-spectrum antibiotics, chemotherapy, steroids, prior surgery, abdominal surgery, central venous catheter (CVC) at the time of diagnosis, parenteral nutrition, among others [1,6], identification of the *Candida* species isolated in blood cultures, Pitt score as the clinical severity index [23], antifungals prescribed, time to treatment initiation, time to CVC removal, and all-cause mortality at 14 and 30 days from index candidemia. No eligible patients had to be excluded from the study due to missing data.

### 2.2. Definitions

Candidemia was defined as the isolation of any *Candida* species from at least one peripheral blood culture. The date the first positive blood culture was collected was set as the date of index candidemia. Patients were included only once during each episode of candidemia. A new episode was defined if blood cultures were positive for *Candida* species after 30 days or more from index candidemia. Early CVC removal was performed within 48 h from the extraction of the first positive blood culture, whereas late CVC removal occurred after 5 days. Severe cases were defined as those with a Pitt score > 1 [23].

### 2.3. Microbiology

*Candida* isolates were identified at species level in the local laboratory and sent to the Special Mycology Laboratory (LEMI) at the Universidade Federal de São Paulo for confirmation. In the first study period, fungal identification at the core laboratory was based on microscopic morphology on cornmeal Tween 80 agar along with biochemical testing using the ID32C system (BioMérieux SA, Marcy l’Étoile, France). In the second period, all *Candida* isolates were identified by Matrix Assisted Laser Desorption Ionization-Time of Flight Mass Spectrometry (MALDI-TOF MS).

### 2.4. Data Analysis

The data are presented using descriptive statistics. Categorical variables were described as counts (%) and compared using either chi-square, or Fisher’s exact test, as appropriate. Continuous variables with a normal distribution were reported as mean ± standard deviation (SD) and compared using the Student’s *t* test. Those with a non-normal distribution were described as median and interquartile range (IQR) and compared using the Mann–Whitney *U*-test. Statistical significance was set at a two-tailed *p*-value < 0.05. Statistical analyses were performed using SPSS V24 software package (SPSS Inc., Chicago, IL, USA).

### 2.5. Ethics Statement

The authors confirm that the ethical policies of the journal have been adhered to, and that this work has been approved by the institutional ethics committee in Brazil–Comitê de Ética em Pesquisa (CEP) Unifesp, study code 44989021.9.1001.5505.

## 3. Results

### 3.1. Epidemiology

A total of 616 cases of candidemia in adults were analyzed, 369 being from Period I (2010–2011) and 247 from Period II (2017–2018). The baseline characteristics of the population studied are described in Table 1. Briefly, patients from both periods had a similar median age [62 years (18–97) vs. 65 (18–93), *p* = 0.139], and the male proportion was balanced [191 (51.8%) vs. 122 (49.4%), *p* = 0.566]. Despite the trend towards more ICU patients in Period I [199 (53.9%) vs. 114 (46.2%), *p* = 0.059], there was no significant difference in initial clinical severity, with 227 (61.5%) patients with a Pitt score > 1 in Period I vs. 168 (68.0%) in Period II, *p* = 0.104. Patients from later years were more likely to be on chronic dialysis [37 (15.0%) vs. 31 (8.4%), *p* = 0.013], to use non-steroid immunosuppressive drugs [61 (24.7%) vs. 29 (7.9%), *p* < 0.001], and to present over three coexisting comorbidities [72 (29.1%) vs. 60 (16.3%), *p* < 0.001]. In addition, a previous in-hospital admission was more frequent among patients in Period II [99 (40.1%) vs. 79 (21.4%), *p* < 0.001], and the onset of their candidemia episode was usually earlier, with a median of 15 (0–328) days from admission vs. 19 (0–188), *p* = 0.01.

### 3.2. Microbiology

*Candida albicans* remained as the single leading isolated species, although non-*albicans* species together predominated in both periods. No differences were found in species distribution, as demonstrated in Table 2. Additionally, mixed infections with more than one *Candida* species isolated in the same blood culture were observed in six episodes of candidemia, with no significant differences between periods.

### 3.3. Therapeutic Management

General therapeutic practices are presented in Table 3. Over time, echinocandins were prescribed more often [50 (13.6%) vs. 102 (41.3%), *p* = 0.001], but no significant changes were observed in time to initiate antifungal treatment [2 days (0–13) vs. 2 (0–14), *p* = 0.369], nor on the rate of early CVC removal [148/319 (46.4%) vs. 90/185 (48.6%), *p* = 0.644].

There was a notable proportion of candidemic patients with no antifungal therapy prescribed in both periods I and II [87 (23.6%) vs. 43 (17.4%), *p* = 0.070], respectively. The particular reasons that led them to be untreated were not specified in the data collected at the time. Nevertheless, when compared to patients who did receive treatment, these adults were older, with a median age of 67 years (18–95) vs. 63 (18–97), *p* = 0.004, presented with candidemia earlier in the course of admission, within a median of 12 days (0–96) vs. 19 (0–328), *p* < 0.001, were more likely to develop candidemia due to *C. glabrata* [30 (23.1%) vs. 51 (10.5%), *p* < 0.001], and had *C. parapsilosis* less often [18 (13.8%) vs. 113 (23.3%), *p* = 0.022]. Additionally, they died sooner after the first positive blood cultures were drawn, within a median of 2 days (0–306) vs. 14 (0–372), *p* < 0.001. A total of 59 (45.4%) patients were dead within 48 h, whereas only 21 (16.1%) were alive at 30 days from fungemia, even without treatment.

A detailed comparison between candidemia therapeutic practices in ICU vs. non-ICU is presented in Table 4. Briefly, ICU patients had earlier antifungal initiation, within 2 days (0–13) vs. 3 (0–14), *p* = 0.007, treatment with fluconazole was less frequent [126 (40.3) vs. 148 (48.9), *p* = 0.035], and early CVC removal was performed more often 147/283 (51.9%) vs. 91/221 (41.2%), *p* = 0.019.

### 3.4. Clinical Outcomes

No differences were observed in mortality rates at 14 or 30 days from index candidemia, regardless of the unit patients were admitted into, as shown in Table 5.

When patients who did not receive antifungals were excluded, there was a trend towards a higher 14-day mortality rate in the general population of patients in Period II, mainly among non-ICU patients, although not statistically significant, as shown in Table 6.

## 4. Discussion

We performed a comparative retrospective multicenter study to analyse trends in the epidemiology, real-life therapeutic practices, and mortality in a large series of adults with candidemia from public and private tertiary hospitals, diagnosed in two different periods within the last decade in Brazil. Unfortunately, mortality rates were unacceptably high and remained unchanged over the years despite a broader use of echinocandins, probably associated with a clear epidemiological change in the population at risk and suboptimal diagnostic and therapeutic strategies.

Our data show no significant improvements in mortality rates over time in line with other contemporary studies [12,24,25], even when favoring the 14-day analysis as a proxy for attributed mortality. On the contrary, general mortality tended to increase in Period II ranging from 22.5% to 30.4% (*p* = 0.058) at 14 days, especially among non-critical patients, although not statistically significant. When only patients who received antifungal treatment were evaluated, mortality rates became slightly lower, but they were still very similar between periods. Many epidemiological studies in Brazil report even higher 30-day crude mortality rates along periods prior to our work [6,8,9,10,11,12], and among other developing countries [7], reaching up to 76.4% among critical care patients [6], but with better clinical outcomes observed after the introduction and better access to echinocandins as first-line antifungal treatment [6,26,27].

Conversely, despite the natural increase in the use of echinocandins shown in our series, such a positive impact on patient survival was no longer observed, as also described by other authors [12,24], suggesting that the optimization of antifungal choice with echinocandins as initial therapy is crucial and recommended by trials and current guidelines [5,27,28], but no single measure is enough to promote additional progress against excess mortality. Along with this shift in therapeutic practice, the use of amphotericin B and fluconazole decreased, but the latter was still responsible for 36% of antifungal initial therapy in Period II, and no progress was made to start antifungals earlier, what clearly affects patient prognosis [18,19,20,21]. In agreement with our findings, Braga et al., in an epidemiological study of historical trends covering two decades, also reported persistently high mortality rates, even though echinocandins were prescribed more often and even despite antifungals were initiated sooner. However, they also described an increase in the number of severely ill patients, and an alarming percentage of cases that went untreated [12].

In our series, the proportion of patients who did not receive antifungals at all seemed to be decreasing over time, from 23.6% to 17.4% in Period II, although not statistically significant. Likewise, in a recent study investigating the attributed mortality of candidemia in the modern era, there was still around 20% of untreated patients who not only experienced even higher mortality rates, but curiously, most of them were classified as unsuspected cases, in patients presenting the lowest risk of developing candidemia [29]. When evaluating their characteristics in our work, untreated patients were older, developed candidemia earlier after admission, and died sooner, within two days from fungemia in almost half of cases, possibly even before the medical staff was aware of the diagnosis. No specific information to justify the reasons for not treating such patients could be obtained. Yet, although some of them were probably too ill to respond to any treatment due to rapid clinical deterioration, efforts should be made to lower the number of untreated eligible patients by detecting infection before it is too late in the disease course [30,31].

Furthermore, our untreated patients were more likely to have candidemia due to *C. glabrata*, as previously observed in another historical trend cohort [12]. This could be at least partially explained by the higher prevalence of *C. glabrata* among older patients [32], but also due to its longer time to grow in culture media [33], consequently delaying diagnosis and treatment. Thus, aside from continuous medical education to guarantee an adequate level of early suspicion of candidemia even in lower-risk patients [29,34] and antifungal stewardship programs to assure rapid and effective antifungal initiation [35], investments on improving and providing more sensitive diagnostic methods with a faster turnaround time are urgently needed [30,31].

Regarding epidemiological trends of the population at risk, our results demonstrate that the aging quality and patient complexity of those who develop candidemia have changed, what could have outbalanced potential advances in candidemia management and general patient care, as previously suggested by other authors [12,24]. Even though the median age and clinical severity score between periods were similar, patients diagnosed in later years have accumulated more comorbidities, were more likely to be on chronic dialysis and on immunosuppressives, besides being exposed to more hospital admissions prior to the candidemia episode, acquiring more risk factors to develop candidemia, and leading to potential colonization of multidrug-resistant isolates and eventual subsequent difficult-to-treat bacterial coinfections [36]. Therefore, these patients could be more fragile and vulnerable to either fail to respond to adequate therapeutical efforts, or to develop aggravating complications accompanying the candidemia episode, increasing overall mortality rates [37].

Nevertheless, in a previous study from our group aiming to better understand mortality disparity between countries [22], candidemic patients from Spain presented significantly lower mortality rates at 14 and at 30 days, despite being older than Brazilian patients, but antifungal initiation and CVC withdrawal were performed notably faster, and the rate of untreated patients was kept as low as 7.4%. In contrast, the present study shows the proportion of early CVC removal remained under 50%, probably leaving room for improvements. We understand CVC removal is not always possible due to safety reasons, disease severity or specific clinical conditions, and should be individualized according to current guidelines [5,13]. Yet, many authors support the concept of CVC removal as one of the main pillars for effective and faster fungal eradication that should be attempted and prioritized for better prognosis [18,21,22,30,38,39], while others emphasize its exclusive benefit either when the CVC is the primary source of infection [19], or among patients with sepsis and septic shock [25].

Finally, when we compared therapeutic management between ICU and non-ICU patients, those from outside of intensive care units had a significant delay in time to initiate antifungal and CVC removal, reflecting a possible lower grade of suspicion on the diagnosis of candidemia, and an underestimation of the importance of early interventions regardless of patients’ initial clinical severity, especially in settings that were once “atypical” for candidemia. This has been described by other groups, mainly in internal medicine wards, and continues to be a current challenge [38,39,40].

Our study has some limitations. Although the data were collected prospectively set off by routine laboratory surveillance, the analyses were made retrospectively based on the variables available for all eleven centers in both periods. Consequently, a few important aspects that could help us better understand such persistent high mortality rates and epidemiological trends were not evaluated, such as the incidence rates of candidemia, data on bacterial coinfections that could influence patient outcome, antifungal susceptibility patterns, detailed information on catheter management in patients with more than one catheter, as well as time to blood culture positivity, time until notification of the medical staff, availability or performance of infectious diseases specialists in stewardship roles, and structural data. Nevertheless, our study provides valuable information from a large multicenter series of candidemia from public and private tertiary hospitals that exposes current challenges in a developing country, reflecting that monitoring trends may help shaping better practical management strategies that should be individualized, supervised, and adapted over time.

In conclusion, adults with candidemia continue to experience exceedingly high mortality rates despite advances in therapeutic practices with a broader use of echinocandins and extensive published knowledge on timely therapeutic cornerstones that evidently impact patient prognosis [5,21,22,25,30], possibly due to the rising complexity of aging patients in later years, combined with a persistent delay in culture-based diagnosis, and a consequent late start of appropriate treatment and source control. Certainly, other challenging aspects unmeasured in this work and common to other developing countries may have played an important role on maintaining elevated mortality rates, including limited resources, operating beyond standard capacity, sub-optimal infection control practices, the unavailability of antifungal stewardship programs to reinforce guidelines, and an overall deficiency of continuous medical education and awareness [7,35]. However, providing universal access to cost-effective and prompt diagnosis before severe clinical deterioration sets in is clearly a key element that should be prioritized to lower rates of untreated eligible patients and trigger earlier therapeutic interventions to improve the prognosis of adult patients with candidemia.

## Figures and Tables

**Table 1:** Baseline characteristics of candidemic adults diagnosed at tertiary hospitals in Brazil during Period I: 2010–2011 vs. Period II: 2017–2018.

Variables	Total (*n* = 616)	Period I (*n* = 369)	Period II (*n* = 247)	*p*-Value
**Demographics**				
Age (years), median (IQR)	61 (18–97)	62 (18–97)	65 (18–93)	0.139
Sex (male), *n* (%)	313 (50.8)	191 (51.8)	122 (49.4)	0.566
Previous in-hospital admission	178 (28.9)	79 (21.4)	99 (40.1)	**<0.001**
ICU admission at diagnosis	313 (50.8)	199 (53.9)	114 (46.2)	0.059
Time from admission to index candidemia, median in days (IQR)	18 (0–328)	19 (0–188)	15 (0–328)	**0.010**
Candidemia within 48 h from admission	81 (13.5)	40 (11.4)	41 (16.6)	0.070
Pitt score > 1	395 (64.1)	227 (61.5)	168 (68.0)	0.104
**Comorbidities, *n* (%)**				
Cardiovascular disease	146 (23.7)	103 (27.9)	43 (17.4)	**0.001**
Pulmonary disease	114 (18.5)	80 (21.7)	34 (13.8)	**0.002**
Liver disease	87 (14.1)	56 (15.2)	31 (12.6)	0.056
Diabetes mellitus	165 (26.8)	96 (26.0)	69 (28.0)	0.533
Insulin-dependent diabetes	107 (17.4)	74 (20.1)	33 (13.4)	0.180
Auto-immune disease	29 (4.7)	17 (4.6)	12 (4.9)	0.162
Kidney failure *	254 (41.2)	143 (38.8)	111 (44.9)	0.133
Chronic dialysis	68 (11.0)	31 (8.4)	37 (15.0)	**0.013**
Neurological disease	150 (24.4)	99 (26.8)	51 (20.6)	0.610
Solid cancer	164 (26.6)	94 (25.5)	70 (28.3)	0.457
Hematological cancer	44 (7.1)	26 (7.0)	18 (7.3)	1
Solid organ transplant	45 (7.3)	30 (8.1)	15 (6.1)	0.971
Three or more comorbidities	132 (21.4)	60 (16.3)	72 (29.1)	**<0.001**
**Associated conditions, *n* (%)**				
Chemotherapy	43 (7.0)	28 (7.6)	15 (6.1)	0.521
Neutropenia (<500 cells/μL)	33 (5.3)	17 (4.6)	16 (6.5)	0.309
Surgery	308 (50.0)	195 (52.8)	113 (45.7)	0.100
Abdominal surgery	163 (26.5)	94 (25.5)	69 (27.9)	0.515
Total parenteral nutrition	138 (22.4)	83 (22.5)	55 (22.4)	0.689
CVC at place	514 (83.4)	320 (86.7)	194 (78.5)	**0.008**
Antibiotic use	543 (88.1)	342 (92.7)	201 (81.4)	**<0.001**
Antifungal use	121 (19.6)	86 (23.3)	35 (14.2)	**0.001**
Corticosteroids	208 (33.8)	148 (40.1)	60 (24.3)	**<0.001**
Other immunosuppressive drugs	90 (14.6)	29 (7.9)	61 (24.7)	**<0.001**

ICU: intensive care unit; CVC: central venous catheter. * Either previous kidney failure or at the moment of candidemia.

**Table 2:** Microbiological characteristics of 616 adult patients with candidemia diagnosed at tertiary hospitals in Brazil during Period I: 2010–2011 vs. Period II: 2017–2018.

Variables	Total (*n* = 616)	Period I (*n* = 369)	Period II (*n* = 247)	*p*-Value
***Candida* species, *n* (%)**				
*C. albicans*	243 (39.4)	143 (38.8)	100 (40.5)	0.675
*C. parapsilosis*	131 (21.3)	75 (20.3)	56 (22.7)	0.547
*C. tropicalis*	112 (18.2)	70 (19.0)	42 (17.0)	0.594
*C. glabrata*	81 (13.1)	49 (13.3)	32 (13.0)	1
*C. krusei*	25 (4.1)	17 (4.6)	8 (3.2)	0.417
*C. guilliermondii*	5 (0.8)	4 (1.1)	1 (0.4)	0.421
Other *	19 (3.1)	11 (3.0)	8 (3.2)	1

* *Candida* species not previously listed, or mixed infections with more than one *Candida* species.

**Table 3:** Overall therapeutic management of 616 adults with candidemia diagnosed at tertiary hospitals in Brazil during Period I: 2010–2011 vs. Period II: 2017–2018.

Variables	Period I (*n* = 369)	Period II (*n* = 247)	*p*-Value
**Antifungal therapy, *n* (%)**			
No antifungals prescribed	87 (23.6)	43 (17.4)	0.070
Echinocandins	50 (13.6)	102 (41.3)	**<0.001**
Fluconazole	185 (50.1)	89 (36.0)	**0.001**
Amphotericin B	47 (12.7)	13 (5.3)	**0.002**
Treatment switch	78 (21.1)	65 (27.9)	0.062
Time to initial treatment, median in days (IQR)	2 (0–13)	2 (0–14)	0.369
**Infection source control, *n* (%)**			
Early CVC removal *	148/319 (46.4)	90/185 (48.6)	0.644
Late CVC removal **	69/319 (21.6)	39/185 (21.1)	0.911
Time to CVC removal, median in days (IQR)	3 (0–38)	3 (0–41)	0.850

CVC: central venous catheter. * Within 48 h from the extraction of the first positive blood culture. ** After 5 days from the extraction of the first positive blood culture.

**Table 4:** Comparison of ICU vs. non-ICU therapeutic management of candidemia in 616 adult patients.

Variables	ICU (*n* = 313)	Non-ICU (*n* = 303)	*p*-Value
**Antifungal therapy, *n* (%)**			
No antifungals prescribed	63 (20.1)	67 (22.1)	0.555
Echinocandins	81 (25.9)	71 (23.4)	0.513
Fluconazole	126 (40.3)	148 (48.9)	**0.035**
Amphotericin B	43 (13.7)	17 (5.6)	**0.001**
Treatment switch	75 (24.4)	68 (23.1)	0.703
Time to initial treatment, median in days (IQR)	2 (0–13)	3 (0–14)	**0.007**
**Infection source control, *n* (%)**			
Early CVC removal *	147/283 (51.9)	91/221 (41.2)	**0.019**
Late CVC removal **	53/283 (18.7)	55/221 (24.9)	0.102
Time to CVC removal, median in days (IQR)	2 (0–38)	3 (0–41)	**0.010**

ICU: intensive care unit; CVC: central venous catheter. * Within 48 h from the extraction of the first positive blood culture. ** After 5 days from the extraction of the first positive blood culture.

**Table 5:** Clinical outcomes of adults with candidemia diagnosed at tertiary hospitals in Brazil during Period I: 2010–2011 vs. Period II: 2017–2018 *.

Outcomes, *n* (%)	Period I	Period II	*p*-Value
**General**			
- 14-day mortality	123/366 (33.6)	93/247 (37.7)	0.343
- 30-day mortality	188/366 (51.4)	120/247 (48.6)	0.511
**Intensive care unit**			
- 14-day mortality	87/198 (43.9)	56/114 (49.1)	0.410
- 30-day mortality	122/198 (61.6)	71/114 (62.3)	1
**Non-ICU**			
- 14-day mortality	36/168 (21.4)	37/133 (27.8)	0.224
- 30-day mortality	66/168 (39.3)	49/133 (36.8)	0.721

ICU: intensive care unit; * Mortality data were not available for three patients due to transfer to other hospitals.

**Table 6:** Clinical outcomes of adults with candidemia diagnosed at tertiary hospitals in Brazil during Period I: 2010–2011 vs. Period II: 2017–2018 * who received antifungal treatment.

Outcomes, *n* (%)	Period I	Period II	*p*-Value
**General**			
- 14-day mortality	63/280 (22.5)	62/204 (30.4)	0.058
- 30-day mortality	115/280 (41.1)	84/204 (41.2)	1
**Intensive care unit**			
- 14-day mortality	48/151 (31.8)	40/98 (40.8)	0.175
- 30-day mortality	77/151 (51.0)	55/98 (56.1)	0.439
**Non-ICU**			
- 14-day mortality	15/129 (11.6)	22/106 (20.8)	0.072
- 30-day mortality	38/129 (29.5)	29/106 (27.4)	0.773

ICU: intensive care unit; * Mortality data were not available for two patients among those who received treatment due to transfer to other hospitals.

## Data Availability

Anonymized data may be available only upon well detailed and pertinent request due to privacy or ethical restrictions. Please contact the corresponding authors.
